# Decreased virulence of a uropathogenic *Escherichiacoli pst* mutant is attributed to the repression of Type 1 fimbriae

**Published:** 2011-01-10

**Authors:** S Crépin, S Houle, J Harel, CM Dozois

**Affiliations:** 1 INRS-Institut Armand-Frappier, 531 boul. des Prairies, Laval, Québec, Canada H7V 1B7; 2 Centre de Recherche en Infectiologie Porcine (CRIP), Université de Montréal, Faculté de Médecine Vétérinaire, Saint-Hyacinthe, Québec, Canada J2S 7C6; 3 Groupe de Recherche sur les Maladies Infectieuses du Porc (GREMIP), Université de Montréal, Faculté de Médecine Vétérinaire, Saint-Hyacinthe, Québec, Canada J2S 7C6

## 

Extra-intestinal pathogenic *E. coli* cause urinary tract infections (UTIs), newborn meningitis, abdominal sepsis and septicemia. UTIs affect millions of women annually, and result in significant health care costs and morbidity worldwide. Uropathogenic *E*. *coli* (UPEC) is the predominant urinary tract pathogen, causing up to 85% of UTIs. Despite appropriate therapy, recurrent episode of UTI are common and bacterial strains are increasingly more resistant to many currently used antimicrobial agents. The *pstSCAB*-*phoU* operon encodes the phosphate specific transport system (Pst) and belongs to the Pho regulon, which is regulated by the two-component regulatory system (TCRS) PhoBR. Inactivation of the Pst system in *E*. *coli* and other bacteria leads to constitutive activation of the Pho regulon, perturbations in cellular adaptation, and decreased virulence. The role of the Pst system in uropathogenic *E. coli* (UPEC) was assessed by deleting the *pstSCA* genes in UPEC strain CFT073. In competitive (co-challenge) and single-strain infections, the *pst* mutant was attenuated for colonization of both the bladder and kidneys of CBA/J mice and was impaired for production of Type 1 fimbriae. Type 1 fimbriae are essential for UPEC virulence and their phase-variable expression is positively and negatively regulated by FimB and FimE, respectively. *In vitro*, in LB broth and human urine, repression of the *fim* structural gene *fimA* in the *pst* mutant correlated with increased orientation of the *fim* promoter in the OFF-position. *In vivo*, down-regulation of *fimA* in CFT073 Δ*pst* correlated with the up-regulation of *fimE*. To confirm the specific role of repression of *fim* expression by the *pst* mutant during UTI, *fim* phase locked-ON *pst* derivatives of the *pst* mutant and WT CFT073 strains were constructed. Compared to the *pst* mutant, the *fim* phase locked-ON *pst* derivative demonstrated a significant gain in colonization of the bladder, that was similar to that of CFT073 WT and CFT073 *fim* locked-ON strains. As Type 1 fimbriae are important for UPEC virulence, by promoting adhesion, our results suggest that the reduced bladder colonization by the *pst* mutant during UTI is predominantly attributed to down-regulation of these fimbriae. Since the Pho regulon is controlled by the TCRS PhoBR, molecules inducing the expression of the Pho regulon through inactivation of Pst or activation of PhoBR could potentially impair UPEC virulence by inhibiting colonization and the infection cycle, which is dependent on expression of type 1 fimbrial adhesins.

